# Does Marriage Really Matter to Health? Intra- and Inter-Country Evidence from China, Japan, Taiwan, and the Republic of Korea

**DOI:** 10.1371/journal.pone.0104868

**Published:** 2014-08-12

**Authors:** Woojin Chung, Roeul Kim

**Affiliations:** 1 Department of Health Policy and Management, Graduate School of Public Health, Yonsei University, Seoul, Korea; 2 Institute of Health Services Research, Yonsei University, Seoul, Korea; 3 Department of Public Health, Graduate School, Yonsei University, Seoul, Korea; Alberta Provincial Laboratory for Public Health/University of Alberta, Canada

## Abstract

**Background:**

The health benefits of marriage have been demonstrated mainly by studies on Western populations. This study aims to test whether the benefits are also valid in East Asian populations.

**Methodology/Principal Findings:**

Individuals (n = 8,538) from China, Japan, Taiwan, and the Republic of Korea were sampled from the 2006 East Asian Social Survey. The association between self-rated health status and two marriage-related independent variables was analyzed using multivariate logistic regression models. In a two-level analysis for individuals from all countries, married individuals were more likely to report very good or good health compared to their never-married counterparts [odds ratio (OR) 1.56; 95% confidence interval (95% CI) 1.16−2.10]. However, the addition of marital satisfaction disintegrated the significant association of marriage with self-rated health. Married individuals in satisfying marriages were more likely to report very good or good health compared with never-married individuals (OR 1.85; 95% CI 1.37−2.50). In contrast, married individuals in dissatisfying marriages were as likely to report very good or good health as never-married individuals (OR 0.78; 95% CI 0.50−1.24). In a one-level analysis for each country, the importance of marital satisfaction varied greatly across countries. Unlike in other countries, in Japan, married individuals in dissatisfying marriages were about half as likely to report very good or good health as never-married individuals (OR 0.51, 95% CI 0.31−0.83), thereby showing no significant benefits from marriage with regard to self-rated health.

**Conclusion/Significance:**

The present study of East Asian countries suggests that marital satisfaction is of greater importance in determining self-rated health than marriage itself, and that the importance of marital satisfaction varies across countries. Further research is required to better understand the relationship between marital satisfaction and self-rated health in different socio-cultural settings, and to establish effective social policies aiming at improving public health.

## Introduction

Researchers have found that various factors affect individual health [1]–[3]. In particular, many studies have focused on the role of marriage, suggesting that marriage is associated with better health [4]–[7]. However, the relationship between marriage and health requires further investigation for several reasons.

First, along with marriage itself, marital satisfaction may be important for health, as shown in previous studies [8], [9]. If this factor is ignored, researchers may fail to understand the health differential between unmarried and married persons. Nonetheless, only a few researchers have paid attention to the variable of marital satisfaction [6], [7], [10], and even their findings are difficult to generalize owing to limitations such as small sample sizes or the use of non-random sampling.

Second, in Asian countries, the association between marriage and health may differ from that found in Western countries, owing to their different socio-cultural characteristics. Past studies on this topic have focused mainly on Western countries, including the U.S. [4], [7], [10], [11], England [12]–[14], Canada [6], [15], Finland [16], Sweden [17], and several other European countries [18], [19].

Finally, the association between marriage and health has not been examined through either a nationwide analysis or an inter-country study. An inter-country study performed in a coordinated research setting is expected to produce evidence that is more generalizable.

Therefore, to better understand the association between marriage and individual health, the present study addresses the three points just mentioned. We analyzed a dataset from the 2006 East Asian Social Survey (EASS), which comprises individuals from China, Japan, Taiwan, and the Republic of Korea (hereafter, Korea), both separately for each country and as a whole.

## Methods

### Data Source and Study Sample

Data were derived from the EASS. The EASS is an East Asian version of the European Social Survey, which has been administered in over 30 countries in Europe. In the EASS, China, Japan, Taiwan, and Korea shared a common module from a General Social Survey-type questionnaire, and each country implemented a nationally representative sample survey. Samples were selected using a multistage stratified random sampling method [20]. The survey methodology was described previously in an online report (http://www.eassda.org/).

In the present study, we used the 2006 EASS dataset, which drew on in-person interviews conducted from June to December 2006 in each country. Out of 8,842 individuals aged 20 years and over in total, we excluded 304 (3.43%) due to missing values, but we encountered no significant differences between the datasets before and after the exclusion (*P*<0.775 for gender; *P*<0.769 for age). Finally, we analyzed data from 8,538 individuals, consisting of 3,054 individuals in China, 1,982 in Japan, 1,981 in Taiwan and 1,521 in Korea.

The EASS data archive provides publicly available data from respondents whose identities are undisclosed. Verbal informed consent was obtained from all participants due to the limited time for survey interviews, and waivers of written consent were authorized by an ethics committee. Ethical approval for this study was granted by the institutional review board of the Graduate School of Public Health, Yonsei University, Seoul, Korea.

### Measures and Variables

The dependent variable was obtained from an individual’s self-rated health. Each individual was asked ‘How would you rate your health?’ and was prompted to answer on a 5-point scale. For analytical simplicity, a dichotomous health variable was constructed such that ‘1’ indicated that the individual’s answer was very good or good, and ‘0’ indicated otherwise.

Two marriage-related variables were separately considered as independent variables: the marital status variable and the marital status and satisfaction variable. For the marital status variable, individuals were divided into three states: (a) married, (b) never-married, and (c) married but single (widowed, divorced, or separated). We removed unmarried individuals cohabitating with their partners because this category included only 22 individuals (0.24%).

To construct the marital status and satisfaction variable, we used additional information provided by each married individual. During the survey, a married individual was asked, ‘All things considered, how would you describe your marriage? Would you say that you are very satisfied or dissatisfied with your marriage?’ and was encouraged to answer on a *5*-point scale. We grouped these answers into three states: (a) married and satisfied, (b) married and average, and (c) married and dissatisfied. Combining these three groups with the remaining two groups in the marital status variable, we set five new categories of the marital status and satisfaction variable: (a) never-married, (b) married but single, (c) married and satisfied, (d) married and average, and (e) married and dissatisfied.

Covariates included various socio-demographic characteristics: gender, age, education, self-rated social class, employment, and religion. Individuals were divided into five age groups: 20−29, 30−39, 40−49, 50−59, and ≥60 years. Education level was categorized into four groups: lowest (no or above lowest qualification), low (higher secondary completed), high (above higher secondary level), and highest (university degree completed). To ascribe self-rated social class, an individual was asked ‘In our society there are groups that tend to be towards the top as well as groups towards the bottom. Where would you put yourself on this scale?’ Available choices were numerical on a 10-point scale in Taiwan, Japan and Korea and on a 5-point scale in China. We converted the 10-point scale into a 5-point scale to be consistent with one another and obtained four categories of a self-assessed social class variable; lowest, low, high, and highest. According to employment status, individuals were divided into two groups: employed and not employed (unemployed, retired, permanently disabled and out of the labor force, students, and housewives). Three groups were constructed based on religion: Christian, Buddhist, and other (Muslim, atheist, agnostic, and other religions).

## Analytic Procedures

For each independent variable, we performed a four-fold analysis. First, we compared the characteristics of the respondents for each country. Second, using a *χ^2^* test, we examined the differences in the proportions of individuals reporting very good or good health for each characteristic. Any characteristic that was significantly related to reporting very good or good health at the 5% level was selected for a multivariate logistic analysis. Third, we pooled individuals from all countries and employed a two-level multivariate logistic analysis, as the individuals in the sample were likely to be correlated within the same country. Finally, in order to draw country-specific findings, we performed a one-level multivariate logistic analysis for each country. For every multivariate logistic analysis, we presented the adjusted odds ratios (ORs) with 95% confidence intervals (CIs) [21]. Values of *P*<0.05 were considered to be statistically significant. All analyses were conducted using SAS version 9.2 (SAS Institute, Cary, NC).

## Results

Out of the entire study population, 65.00% of individuals reported very good or good health from the four countries. China and Taiwan showed relatively high proportions of individuals reporting very good or good health (75.54% and 72.64%, respectively), with lower proportions in Japan and Korea (48.69% and 55.16%, respectively) (Table 1). The proportion of never-married individuals was highest in Taiwan (26.25%) and lowest in China (10.58%). China had the highest proportion of married individuals who were satisfied with marriage (68.86%), whereas Korea had the lowest proportion (40.04%). Regarding the covariates, the countries with the highest proportion of individuals with a particular characteristic were: Japan for individuals aged 60 years and over (37.24%), Korea for individuals who completed a university degree (27.88%), Taiwan for individuals who reported belonging to the highest level of social class (22.46%), China for employed individuals (90.18%), and Korea for individuals affiliated with Christianity (31.69%).

**Table 1:** Sample characteristics for each country.

	China (n = 3054)		Japan (n = 1982)		Taiwan (n = 1981)		Korea (n = 1521)	
	n	(%)	n	(%)	N	(%)	n	(%)
**Self-rated health**								
Very good or good health	2307	(75.54)	965	(48.69)	1439	(72.64)	839	(55.16)
Fair, bad or very bad	747	(24.46)	1017	(51.31)	542	(27.36)	682	(44.84)
**Marital status factors**								
Never-married	323	(10.58)	305	(15.39)	520	(26.25)	326	(21.43)
Married but single^a^	184	(6.02)	257	(12.97)	215	(10.85)	157	(10.32)
Married	2547	(83.40)	1420	(71.64)	1246	(62.90)	1038	(68.24)
**Marital status and satisfaction factors**								
Never-married	323	(10.58)	305	(15.39)	520	(26.25)	326	(21.43)
Married but single^a^	184	(6.02)	257	(12.97)	215	(10.85)	157	(10.32)
Married and satisfied	2103	(68.86)	864	(43.59)	1035	(52.25)	609	(40.04)
Married and average	355	(11.62)	432	(21.80)	148	(7.47)	335	(22.02)
Married and dissatisfied	89	(2.91)	124	(6.26)	63	(3.18)	94	(6.18)
**Socio-demographic characteristics**								
Gender								
Female	1675	(54.85)	1088	(54.89)	978	(49.37)	833	(54.77)
Male	1379	(45.15)	894	(45.11)	1003	(50.63)	688	(45.23)
Age, y								
≤29	555	(18.17)	217	(10.95)	412	(20.80)	277	(18.21)
30–39	746	(24.43)	318	(16.04)	392	(19.79)	382	(25.12)
40–49	722	(23.64)	310	(15.64)	438	(22.11)	413	(27.15)
50–59	642	(21.02)	399	(20.13)	346	(17.47)	192	(12.62)
≥60	389	(12.74)	738	(37.24)	393	(19.84)	257	(16.90)
Education								
Lowest	1917	(62.77)	367	(18.52)	786	(39.68)	336	(22.09)
Low	705	(23.08)	984	(49.65)	485	(24.48)	461	(30.31)
High	288	(9.43)	234	(11.81)	334	(16.86)	300	(19.72)
Highest	144	(4.72)	397	(20.03)	376	(18.98)	424	(27.88)
Self-rated social class								
Lowest	1231	(40.31)	147	(7.42)	118	(5.96)	120	(7.89)
Low	907	(29.70)	447	(22.55)	268	(13.53)	497	(32.68)
High	839	(27.47)	1053	(53.13)	1150	(58.05)	681	(44.77)
Highest	77	(2.52)	335	(16.90)	445	(22.46)	223	(14.66)
Employment								
Not employed^b^	300	(9.82)	757	(38.19)	666	(33.62)	603	(39.64)
Employed	2754	(90.18)	1225	(61.81)	1315	(66.38)	918	(60.36)
Religion								
Christian	59	(1.93)	31	(1.56)	95	(4.80)	482	(31.69)
Buddhist	184	(6.02)	507	(25.58)	455	(22.97)	428	(28.14)
Other	2811	(92.04)	1444	(72.86)	1431	(72.24)	611	(40.17)

The sample size was n = 8538.

a ‘Married but single’ includes widowed, divorced, and separated.

b ‘Not employed’ includes the unemployed, the retired, the permanently disabled out of labor force, students, and housewives.

Table 2 presents the unadjusted association of each characteristic with reporting very good or good health for each country. Considering only marital status, never-married individuals showed the highest proportion in reporting very good or good health in every country. However, when considering marital status and satisfaction, the group with the highest proportion of individuals reporting very good or good health in both Japan and Korea was married individuals who were satisfied with marriage. Moreover, every covariate was significantly associated with reporting very good or good health for at least one country.

**Table 2:** Unadjusted association of each characteristic with reporting very good or good health for each country.

	China (n = 3054)			Japan (n = 1982)			Taiwan (n = 1981)			Korea (n = 1521)		
	n	(%)	P-value	n	(%)	P-value	n	(%)	P-value	n	(%)	P-value
**Marital status factors**												
Marital status			<0.001			0.005			<0.001			<0.001
Never married	323	(85.14)		305	(55.41)		520	(79.62)		326	(63.50)	
Married but single^a^	184	(60.33)		257	(41.63)		215	(58.14)		157	(27.39)	
Married	2547	(75.42)		1420	(48.52)		1246	(72.23)		1038	(56.74)	
**Marital status and satisfaction factors**												
Marital status and satisfaction			<0.001			<0.001			<0.001			<0.001
Never-married	323	(85.14)		305	(55.41)		520	(79.62)		326	(63.50)	
Married but single^a^	184	(60.33)		257	(41.63)		215	(58.14)		157	(27.39)	
Married and satisfied	2103	(77.75)		864	(56.94)		1035	(74.78)		609	(65.52)	
Married and average	355	(65.63)		432	(36.81)		148	(61.49)		335	(45.97)	
Married and dissatisfied	89	(59.55)		124	(30.65)		63	(55.56)		94	(38.30)	
**Socio-demographic characteristics**												
Gender			<0.001			0.511			0.006			<0.001
Female	1675	(73.01)		1088	(49.36)		978	(69.84)		833	(47.90)	
Male	1379	(78.61)		894	(47.87)		1003	(75.37)		688	(63.95)	
Age, y			<0.001			<0.001			<0.001			<0.001
≤29	555	(89.37)		217	(60.83)		412	(83.01)		277	(67.51)	
30–39	746	(83.91)		318	(58.18)		392	(81.38)		382	(61.26)	
40–49	722	(77.42)		310	(48.06)		438	(76.26)		413	(59.56)	
50–59	642	(65.73)		399	(45.61)		346	(66.47)		192	(46.35)	
≥60	389	(52.44)		738	(42.95)		393	(54.45)		257	(32.30)	
Education			<0.001			<0.001			<0.001			<0.001
Lowest	1917	(71.94)		367	(38.15)		786	(60.31)		336	(30.95)	
Low	705	(80.14)		984	(47.87)		485	(76.29)		461	(52.71)	
High	288	(81.94)		234	(59.40)		334	(83.83)		300	(67.67)	
Highest	144	(88.19)		397	(54.16)		376	(83.78)		424	(68.16)	
Self-rated social class			<0.001			<0.001			<0.001			<0.001
Lowest	1231	(69.86)		147	(34.69)		118	(37.29)		120	(31.67)	
Low	907	(77.51)		447	(43.62)		268	(67.54)		497	(50.91)	
High	839	(81.17)		1053	(50.24)		1150	(74.09)		681	(58.59)	
Highest	77	(81.82)		335	(56.72)		445	(81.35)		223	(66.82)	
Employment			0.074			<0.001			<0.001			<0.001
Not employed^b^	300	(71.33)		757	(43.46)		666	(60.21)		603	(46.43)	
Employed	2754	(76.00)		1225	(51.92)		1315	(78.94)		918	(60.89)	
Religion			0.051			0.665			0.008			0.033
Christian	59	(67.80)		31	(54.84)		95	(68.42)		482	(51.45)	
Buddhist	184	(69.57)		507	(49.70)		455	(67.47)		428	(53.74)	
Other	2811	(76.09)		1444	(48.20)		1431	(74.56)		611	(59.08)	

P-values were based on the χ^2^ test; The sample size was n = 8538.

a ‘Married but single’ includes widowed, divorced, and separated.

b ‘Not employed’ includes the unemployed, the retired, the permanently disabled out of labor force, students, and housewives.

Using a two-level multivariate logistic analysis, we obtained the adjusted ORs of reporting very good or good health for individuals in the four countries as a whole (Table 3). The inter-class correlation coefficient from the two-level analysis without explanatory variables was 0.0697, which suggests that there was some degree of clustering among individuals within the same country. The ratios of the generalized chi-square statistic and its degrees of freedom were both 1, suggesting that variability in the data was properly modeled without any residual over-dispersion.

**Table 3:** Adjusted associations of marriage with reporting very good or good health for China, Japan, Taiwan, and Korea (a two-level multivariate logistic analysis).^a^

	OR	(95% CI)	p-value
**Marital status factors**			
Never-married (ref)	1.00		
Married but single^b^	1.27	(0.86−1.88)	0.144
Married	1.56	(1.16−2.10)	0.017
Fit statistics			
Generalized χ^2^		8522.65	
Generalized χ^2^/DF		1.00	
**Marital status and satisfaction factors**			
Never-married (ref)	1.00		
Married but single^b^	1.22	(0.83−1.79)	0.207
Married and satisfied	1.85	(1.37−2.50)	0.007
Married and average	1.04	(0.74−1.46)	0.752
Married and dissatisfied	0.78	(0.50−1.24)	0.191
Fit statistics			
Generalized χ^2^		8510.70	
Generalized χ^2^/DF		1.00	

CI = confidence interval; OR = odds ratio; The sample size was n = 8538.

a The model was adjusted for gender, age, education, self-rated social class, employment, and religion.

b ‘Married but single’ included widowed, divorced, and separated.

When marital status was used as an independent variable, compared with never-married individuals, married individuals were more likely to rate their health as very good or good (OR 1.56 95% CI 1.16–2.10). Meanwhile, when the marital status and satisfaction variable was used as an independent variable, married individuals who were satisfied with marriage were more likely to report very good or good health compared to their never-married counterparts (OR 1.85, 95% CI 1.37−2.50). In contrast, married individuals who were dissatisfied with marriage were less likely to rate their health as very good or good compared to never-married individuals, although the difference was insignificant (OR 0.78; 95% CI 0.50−1.24).

Using a one-level multivariate logistic analysis, we obtained the adjusted associations of each marriage-related variable with reporting very good or good health for each country (Table 4). The results showed no evidence of a lack of goodness-of-fit based on the c-statistic and the Hosmer-Lemeshow statistic. Moreover, no strong multicollinearity was found, as shown by the values of the variance inflation factor, which were lower than 3.5 in each multivariate linear regression analysis. When marital status was used as an independent variable, the group with the highest likelihood of reporting very good or good health was no longer the never-married group, contrary to the unadjusted findings. Married individuals showed the highest likelihood of reporting very good or good health in China (OR 1.72, 95% CI 1.13−2.62), Taiwan (OR 1.49, 95% CI 1.02−2.17), and Korea (OR 1.70, 95% CI 1.13−2.56). In Japan, however, the likelihood of reporting very good or good health was not significantly different between married and never-married individuals.

**Table 4:** Adjusted association of marriage with reporting very good or good health for each country.^a.^

	China (n = 3054)			Japan (n = 1982)			Taiwan (n = 1981)			Korea (n = 1521)		
	OR	(95% CI)	P-value	OR	(95% CI)	P-value	OR	(95% CI)	P-value	OR	(95% CI)	P-value
**Marital status factors**												
Never-married (ref)	1.00			1.00			1.00			1.00		
Married but single^b^	1.32	(0.78−2.25)	0.304	1.04	(0.68−1.59)	0.851	1.37	(0.85−2.22)	0.202	1.01	(0.57−1.78)	0.982
Married	1.72	(1.13−2.62)	0.012	1.14	(0.82−1.58)	0.450	1.49	(1.02−2.17)	0.039	1.70	(1.13−2.56)	0.012
c-statistic	0.698			0.614			0.706			0.702		
H-L test, χ^2^ (p)	6.851 (0.553)			11.091 (0.197)			9.988 (0.266)			9.204 (0.325)		
**Marital status and satisfaction factors**												
Never-married (ref)	1.00			1.00			1.00			1.00		
Married but single^b^	1.29	(0.76−2.19)	0.352	0.98	(0.64−1.49)	0.925	1.34	(0.83−2.16)	0.237	0.90	(0.51−1.60)	0.714
Married and satisfied	1.88	(1.23−2.87)	0.004	1.55	(1.10−2.17)	0.012	1.62	(1.11−2.37)	0.013	2.06	(1.35−3.14)	0.001
Married and average	1.19	(0.74−1.92)	0.471	0.71	(0.49−1.03)	0.070	1.06	(0.64−1.75)	0.814	1.17	(0.74−1.85)	0.503
Married and dissatisfied	0.82	(0.45−1.49)	0.511	0.51	(0.31−0.83)	0.008	0.85	(0.45−1.63)	0.630	0.93	(0.51−1.70)	0.813
c-statistic	0.706			0.651			0.711			0.713		
H-L test, χ^2^ (p)	4.213 (0.837)			6.299 (0.614)			13.156 (0.107)			6.404 (0.602)		

CI = confidence interval; OR = odds ratio; H–L test indicates Hosmer-Lemeshow goodness-of-fit test; The sample size was n = 8538.

a The model was adjusted for gender, age, education, self-rated social class, employment, and religion.

b ‘Married but single’ included widowed, divorced, and separated.

When the marital status and satisfaction variable was used as an independent variable, married individuals who were satisfied with marriage were more likely to report very good or good health than never-married individuals, as shown in China (OR 1.88, 95% CI 1.23−2.87), Japan (OR 1.55, 95% CI 1.10−2.17), Taiwan (OR 1.62, 95% CI 1.11–2.37), and Korea (OR 2.06, 95% CI 1.35–3.14). Meanwhile, with regard to dissatisfaction with marriage, in Japan married individuals who were dissatisfied with their marriage were significantly about half as likely as their never-married counterparts to report their health as very good or good (OR 0.51, 95% CI 0.31−0.83). Individuals in dissatisfying marriages showed, although the difference was neglectable, a lower likelihood of reporting very good or good health than never-married individuals, as shown in China (OR 0.82, 95% CI 0.45−1.49), Taiwan (OR 0.85, 95% CI 0.45−1.63), and Korea (OR 0.93, 95% CI 0.51−1.70).

## Discussion

### Association of Marital Status and Marital Satisfaction with Self-Rated Health

Without taking marital satisfaction into account, many studies on Western populations showed that marriage has a positive influence on individual health [9], [11], [17], [22]–[24]. In a study of 11,112 individuals over a time span of 20 years using data from the Panel Study of Income Dynamic in the U.S., Lillard and Waite [22] found that married individuals had substantially lower risks of death than their unmarried counterparts. In this study, we found that the adjusted association between marital status and self-rated health was significant in all countries surveyed, except for Japan. Moreover, when individuals from all four countries were considered as a whole, this association was found to be statistically significant.

Surprisingly few studies have considered marital status as well as marital satisfaction when comparing unmarried and married people. St John and Montgomery analyzed 1,751 adults aged 65 years and over in Manitoba, Canada from 1991 to 1992 and found that, compared to never-married individuals, married individuals who were dissatisfied with their partner had a higher likelihood of depressive symptoms [6].

In an analysis of 413 women aged 42 to 50 years in 1983 and 1986 from the Pittsburgh Healthy Women Study in the U.S., Troxel et al. found that women who were single or moderately satisfied with marriage did not differ significantly in terms of the risk of developing a metabolic syndrome, whereas women satisfied with their marriage were at lower risk than their unmarried counterparts [10]. To examine the influence of marital status, relationship quality, and network support on ambulatory blood pressure (ABP) and mental health, Holt-Lunstad et al. analyzed data from 204 married and 99 single individuals in the U.S. [7]. They found that married individuals had greater blood pressure dips than unmarried individuals and that better marital quality was associated with lower ABP, lower stress, and less depression. Moreover, they observed that single individuals had lower 24-h and waking ABP compared to those in unhappy marriages.

The few studies that have considered the health effects of both marital status and marital satisfaction are of great value, but they are difficult to generalize due to a range of study limitations, including the non-random nature of the study population [6], [7], [10], small sample size [6], [7], [10], an insufficient number of unmarried people [7], unknown study year and place [7], the absence of comprehensive health outcome measures [6], [7], [10], and limited age ranges [6], [10], gender [10], location [10], and socio-demographic groups [7]. Another limitation is the intra-country nature of these investigations [6], [7], [10]. Furthermore, to the best of our knowledge, no study has researched this issue in any Asian country.

However, in the present study of East Asian countries, which provides more generalized results than in previous studies, we found that marriage benefits with regard to self-rated health were restricted to cases in which individuals maintained a satisfying marriage. As shown in China, Taiwan, and Korea, married individuals reporting that their marriage was average in terms of satisfaction or that they were dissatisfied with their marriage did not display evidence of any marriage benefits on their self-rated health. Even worse, in Japan, married individuals who were dissatisfied with their marriage suffered from a paucity of good self-rated health compared with their never-married counterparts, in that being married did not become significantly associated with self-rated health for these individuals.

### Potential Differences in the Reasons for the Importance of Marital Status and Satisfaction between Western and East Asian Countries

Prior studies of Western countries explained why marriage benefits individual health in terms of two major effects: the ‘marriage selection’ effect and the ‘marriage protection’ effect [25], [26]. First, the marriage selection effect refers to the notion that healthier individuals are more likely to get married than less healthy individuals [14], [27]. For example, individuals with chronic conditions or dangerous or unhealthy lifestyles may have more trouble attracting a spouse compared to healthy and relatively settled individuals [28], [29]. Second, the marriage protection effect refers to marriage itself and how it can reduce mortality and morbidity [22]. For example, through inter-spousal communication, marriage may encourage healthy behaviors, such as visiting doctors [30], and discourage risky behaviors, such as smoking, heavy alcohol use, or illicit drug use [17], [22]. Also, marriage may provide psychological benefits such as reduced stress or improved social ties [22], [31].

However, marriage selectivity may not apply as significantly to Asian societies. Relative to Western countries, marriage is still nearly universal in Asia, for the purposes of being treated as a mature adult and having children [32], [33]. Additionally, divorce is less frequent in Asia owing to cultural reasons, social norms, or a lack of financial independence in women [34]–[36]. Furthermore, marriage tends to be thought of not as an individual-to-individual but as a family-to-family contract in Asian countries, such that many marriage-related decisions are still strongly influenced by parental and relatives’ interventions [35], [37].

Second, inter-spousal communication may not be as strong in Asian countries as it is in Western countries. Traditionally, the husband and wife as a married couple tend to demarcate their household responsibilities in Asian societies; most husbands have been exclusively obliged to procure jobs and make money for their families, whereas most wives have taken full responsibility for completing household chores and raising and educating the children [35], [38], [39]. Particularly, married spouses usually do not talk openly about issues regarding each other’s health, which is often considered as being ill-mannered [39]–[41]. Finally, the degree to which marriage provides psychological benefits may differ between Western and East Asian countries. For example, in Western countries, marriage could likely increase psychological benefits rather than psychological costs, thereby offering a significant, beneficial effect of marriage on health. However, in East Asian countries, psychological benefits due to marriage may be overshadowed by psychological costs. Unfortunately, due to the lack of useful data, we could not compare this difference between Western and East Asian countries. However, it may be presumed that, due to social restrictions upon entry into and exit from marriage as well as the lack of inter-spousal communication regarding health, married individuals in East Asian countries are more likely than their Western counterparts to maintain their unhappy marriages, incurring psychological costs.

### Advantages and Limitations of the Present Study

To the best of our knowledge, the present study is the first to analyze the association between marital status and marital satisfaction with self-rated health, nationwide as well as across countries. Using the 2006 EASS dataset obtained through a coordinated research setting, we examined the four East Asian countries of China, Japan, Taiwan, and Korea, both separately and as a whole. Additionally, we employed a two-level multivariate logistic model to adjust for possible correlations between individuals within the same country.

Despite these advantages, this study still has several limitations. First, the 2006 EASS dataset had relatively low response rates. Valid response rates were 38.5% in China, 59.8% in Japan, 42.0% in Taiwan, and 65.7% in Korea. Nonetheless, the dataset has been shown to be not statistically different from the corresponding national census data in each country [20], [42]. Second, characteristics such as chronic diseases, smoking, and social networks could not be included as covariates owing to the absence of information pertaining to these variables in the dataset. Third, objective health may be a better measure than self-rated health. However, measures of self-rated health have proven to be both reliable and valid health indicators with sufficient variability in a wide range of age groups [43], [44]. However, subjective reports of health status may be confounded by other variables, such as neuroticism or psychological distress, and may not correlate with the underlying pathology [45]. Also, there is some uncertainty about what is being measured when using self-rated health as an health outcome [46]. Nevertheless, self-rated health has been shown to be closely related to the objective health level by many researchers [3], [44], [47], [48]. Self-rated health has reliability and validity for morbidity and mortality [47], [48]. In addition, self-report methods that focus on specific, well-operationalized symptoms are reliably associated with physicians’ diagnoses [49]. Also, self-rated health has been found to be a good predictor of people’s future health care use [47]. Fourth, using a cross-sectional analysis, we could not adjust for the ‘marriage selection’ problem, as discussed earlier. Fifth, many previous studies have found that depressive symptoms are strongly associated with poorer self-rated health [50]–[52]. Moreover, people with depression may be at higher risk of poor marital relations [6], [53], [54]. However, because the 2006 EASS is cross-sectional and did not provide detailed information about depression, we cannot account for this possibility. Further longitudinal studies are necessary to test the directionality of the relationships among depression, self-rated health and marital satisfaction. Finally, it is important to bear in mind that the use of cross-sectional data precludes any definitive causal conclusions about the relationship between marital quality and self-rated health. However, in a recent study of 707 continuously married adults over a 20-year period in the US, Proulx and Snyder-Rivas showed no evidence that changes in self-rated health predicted those in marital status or marital stability over time [55].

## Conclusion

The present study of East Asian countries suggests that marriage benefits with regard to self-rated health are significant, but that these results only apply to individuals in satisfying marriages. The study also suggests that marital satisfaction, rather than marriage itself, gives much detailed explanation of self-rated health. Compared to never-married individuals, married individuals in satisfying marriages generally have better self-rated health, whereas married individuals in dissatisfying marriages may either show no difference in self-rated health, or report worse self-rated health.

However, because this was a cross-sectional study, we could not draw a causal relationship between marital satisfaction and self-rated health. Further longitudinal studies are required to clarify the causal relationship between marital satisfaction and health.

Meanwhile, these conclusions introduce further questions. For example, in which countries is marital satisfaction more important than marriage itself in determining individual health? Moreover, if marital satisfaction is indeed more important than simply marriage, should public health agencies break up unhappy marriages and incentivize single people in order to improve public health? Given these questions, this study highlights the necessity of extensive in-depth research on the importance of marital satisfaction with regard to individual health locally and internationally. Undoubtedly, further research will contribute to a better understanding of the relationship between marriage and individual health and will aid in establishing effective social policies to improve public health.
